# Phases of cortical actomyosin dynamics coupled to the neuroblast polarity cycle

**DOI:** 10.7554/eLife.66574

**Published:** 2021-11-15

**Authors:** Chet Huan Oon, Kenneth E Prehoda

**Affiliations:** 1 Institute of Molecular Biology, Department of Chemistry and Biochemistry, University of Oregon Eugene United States; Whitehead Institute/MIT United States; University of California, Los Angeles United States

**Keywords:** cell polarity, actomyosin, stem cells, differentiation, *D. melanogaster*

## Abstract

The Par complex dynamically polarizes to the apical cortex of asymmetrically dividing *Drosophila* neuroblasts where it directs fate determinant segregation. Previously, we showed that apically directed cortical movements that polarize the Par complex require F-actin (Oon and Prehoda, 2019). Here, we report the discovery of cortical actomyosin dynamics that begin in interphase when the Par complex is cytoplasmic but ultimately become tightly coupled to cortical Par dynamics. Interphase cortical actomyosin dynamics are unoriented and pulsatile but rapidly become sustained and apically-directed in early mitosis when the Par protein aPKC accumulates on the cortex. Apical actomyosin flows drive the coalescence of aPKC into an apical cap that depolarizes in anaphase when the flow reverses direction. Together with the previously characterized role of anaphase flows in specifying daughter cell size asymmetry, our results indicate that multiple phases of cortical actomyosin dynamics regulate asymmetric cell division.

## Introduction

The Par complex polarizes animal cells by excluding specific factors from the Par cortical domain (Lang and Munro, 2017; Venkei and Yamashita, 2018). In *Drosophila* neuroblasts, for example, the Par domain forms at the apical cortex during mitosis where it prevents the accumulation of neuronal fate determinants, effectively restricting them to the basal cortex. The resulting cortical domains are bisected by the cleavage furrow leading to fate determinant segregation into the basal daughter cell where they promote differentiation (Homem and Knoblich, 2012). It was recently discovered that apical Par polarization in the neuroblast is a multistep process in which the complex is initially targeted to the apical hemisphere early in mitosis where it forms a discontinuous meshwork (Kono et al., 2019; Oon and Prehoda, 2019). Cortical Par proteins then move along the cortex toward the apical pole, ultimately leading to formation of an apical cap that is maintained until shortly after anaphase onset (Oon and Prehoda, 2019). Here, we examine how the cortical movements that initiate and potentially maintain neuroblast Par polarity are generated.

An intact actin cytoskeleton is required for the movements that polarize Par proteins to the neuroblast apical cortex, but its role in the polarization process has been unclear. Depolymerization of F-actin causes apical aPKC to spread to the basal cortex (Hannaford et al., 2018; Oon and Prehoda, 2019), prevents aPKC coalescence, and induces disassembly of the apical aPKC cap (Oon and Prehoda, 2019), suggesting that actin filaments are important for both apical polarity initiation and its maintenance. How the actin cytoskeleton participates in polarizing the Par complex in neuroblasts has been unclear, but actomyosin plays a central role in generating the anterior Par cortical domain in the *C. elegans* zygote. Contractions oriented toward the anterior pole transport the Par complex from an evenly distributed state (Illukkumbura et al., 2020; Lang and Munro, 2017). Bulk transport is mediated by advective flows generated by highly dynamic, transient actomyosin accumulations on the cell cortex (Goehring et al., 2011). While cortical movements of actomyosin drive formation of the Par domain in the worm zygote and F-actin is required for neuroblast apical Par polarity, no apically directed cortical actomyosin dynamics have been observed during the neuroblast polarization process, despite extensive examination (Barros et al., 2003; Cabernard et al., 2010; Connell et al., 2011; Koe et al., 2018; Roth et al., 2015; Roubinet et al., 2017; Tsankova et al., 2017). Instead, both F-actin and myosin II have been reported to be cytoplasmic or uniformly cortical in interphase, and apically enriched at metaphase (Barros et al., 2003; Koe et al., 2018; Tsankova et al., 2017), before undergoing cortical flows toward the cleavage furrow that are important for cell size asymmetry (Cabernard et al., 2010; Connell et al., 2011; Roubinet et al., 2017).

The current model for neuroblast actomyosin dynamics is primarily based on the analysis of fixed cells or by imaging a small number of medial sections in live imaging experiments and we recently found that rapid imaging of the full neuroblast volume can reveal dynamic phases of movement that are not detected with other methods (LaFoya and Prehoda, 2021; Oon and Prehoda, 2019). Here, we use rapid full volume imaging to investigate whether cortical actomyosin dynamics are present in neuroblasts when the Par complex undergoes its polarity cycle.

## Results and discussion

### Cortical actin dynamics during the neuroblast polarity cycle

**Figure 1.**
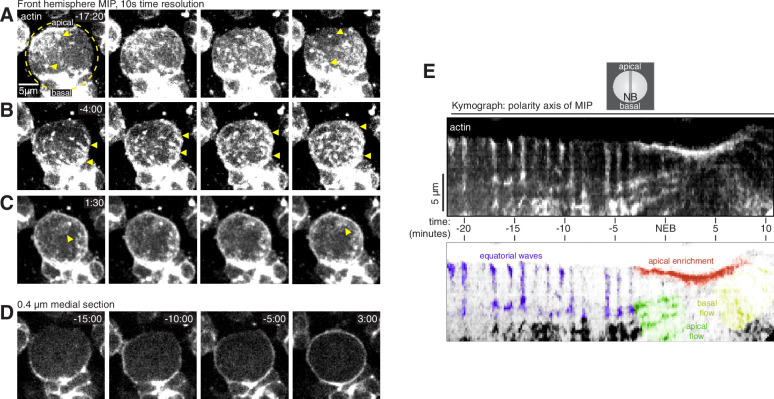
Cortical F-actin dynamics in asymmetrically dividing *Drosophila* larval brain neuroblasts. (**A**) Selected frames from Video 1 showing cortical actin pulses during interphase. mRuby-LifeAct expressed via insc-GAL4/UAS (“actin”) is shown via a maximum intensity projection (MIP) constructed from optical sections through the front hemisphere of the cell. The outline of the neuroblast is shown by a dashed yellow circle. In this example, the pulse moves from the upper left of the cell to the lower right. Arrowheads mark several cortical actin patches. Time (mm:ss) is relative to nuclear envelope breakdown. (**B**) Selected frames from Video 1 as in panel A showing cortical actin moving apically. Arrowheads delineate apical and basal extent of dynamic actin. (**C**) Selected frames from Video 1 as in panel A showing cortical actin enriched on the apical cortex. (**D**) Selected frames from Video 1 showing how actin becomes cortically enriched near nuclear envelope breakdown (NEB). A single 0.4 µM medial section of the cortical actin signal is relatively discontinuous before NEB, with areas of very low actin signal, but becomes more evenly distributed as the cell rounds in mitosis (3:00 time point). (**E**) Kymograph constructed from frames of Video 1 using sections along the apical-basal axis as indicated (NB, neuroblast). A legend with the features in the kymograph depicting the cortical dynamic phases and apical enrichment of actin is included below.

We imaged larval brain neuroblasts expressing an mRuby fusion of the actin sensor LifeAct (mRuby-LA) using spinning disk confocal microscopy. The localization of this sensor in neuroblasts has been reported (Abeysundara et al., 2018; Roubinet et al., 2017), but only during late mitosis. To follow cortical actin dynamics across full asymmetric division cycles, we collected optical sections through the entire neuroblast volume (~40 0.5 µm sections) at 10 s intervals beginning in interphase and through at least one mitosis (Figure 1—figure supplement 1). Maximum intensity projections constructed from these data revealed localized actin enrichments on the cortex, some of which were highly dynamic (Figure 1 and Video 1). We observed three discrete phases of cortical actin dynamics that preceded the previously characterized basally directed flows that occur in late anaphase (Roubinet et al., 2017).

**Figure 1—figure supplement 1.**
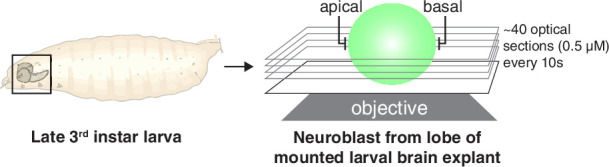
Imaging and analysis scheme for rapid, full volume imaging of *Drosophila* neuroblasts from larval brain explants. Larval brains from 3rd instar larvae were mounted and imaged along the neuroblast polarity axis (‘apical’ and ‘basal’). Optical sections across the full cell volume were acquired every 10–15 s and used to construct maximum intensity projections.

**Video 1.** Actin dynamics in a larval brain neuroblast. The mRuby-Lifeact sensor expressed from the UAS promoter and *insc-GAL4* (expressed in neuroblasts and their progeny) is shown with a maximum intensity projection of the front hemisphere of the cell.

The interphase neuroblast cortex was a mixture of patches of concentrated actin, highly dynamic pulsatile waves that traveled across the entire width of the cell, and areas with little to no detectable actin (Figure 1 and Video 1). Pulsatile movements consisted of irregular patches of actin forming on the cortex and rapidly moving across the surface before disappearing (Figure 1A and E). Concentrated actin patches were relatively static, but sometimes changed size over the course of several minutes. Static patches were mostly unaffected by the pulsatile waves that occasionally passed over them (Figure 1A). Pulses were sporadic in early interphase but became more regular near mitosis, with a new pulse appearing immediately following the completion of the prior one (Figure 1E and Video 1). The direction of the pulses during interphase was highly variable, but often along the cell’s equator (i.e. orthogonal to the polarity/division axis). In general, actin in the interphase cortex was highly discontinuous and included large areas with little to no detectable actin in addition to the patches and dynamic pulses described above (Figure 1D and Video 1). Interphase pulses were correlated with cellular scale morphological deformations in which these areas of low actin signal were displaced away from the cell center while the cortex containing the actin pulse was compressed toward the center of the cell (Figure 1D and Video 1).

Near nuclear envelope breakdown (NEB), the static cortical actin patches began disappearing from the cortex while dynamic cortical actin reoriented toward the apical pole (Figure 1 and Video 1). In contrast to the sporadic and relatively unoriented interphase pulses observed earlier in the cell cycle, the apically directed cortical actin dynamics that began near NEB were highly regular and were apically-directed (Figure 1E and Video 1). This phase of cortical actin dynamics continued until anaphase–consistent with previous descriptions of actin accumulation at the apical cortex throughout metaphase (Barros et al., 2003; Tsankova et al., 2017). Additionally, while the interphase cortex had areas with very little actin, actin was more evenly-distributed following the transition, as was apparent in medial sections (e.g. comparing –15:00 and 3:00 in Figure 1D and Video 1).

Another transition in cortical actin dynamics occurred shortly after anaphase onset when the apically directed cortical actin movements rapidly reversed direction such that the F-actin that had accumulated in the apical hemisphere began to move basally toward the emerging cleavage furrow (Figure 1E and Video 1). The basally directed phase of movement that begins shortly after anaphase onset and includes both actin and myosin II was reported previously (Barros et al., 2003; Roubinet et al., 2017; Tsankova et al., 2017).

### Cortical actin and aPKC dynamics are coupled

**Figure 2.**
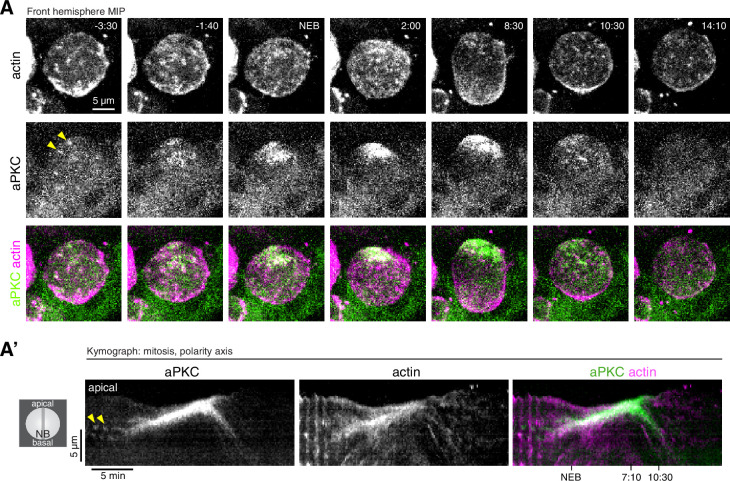
Coordinated actin and aPKC dynamics during the neuroblast polarity cycle.

Previously, we showed that Par polarity proteins undergo complex cortical dynamics during neuroblast asymmetric cell division and that polarity cycle movements require an intact actin cytoskeleton (Oon and Prehoda, 2019). Here, we have found that the cortical actin cytoskeleton is also highly dynamic at points in the cell cycle when Par proteins undergo coordinated cortical movement (Figure 1 and Video 1). Furthermore, the transitions in cortical actin dynamic phases appeared to occur when similar transitions take place in the polarity cycle. We examined the extent to which cortical actin and aPKC dynamics are correlated by simultaneously imaging GFP-aPKC expressed from its endogenous promoter with mRuby-Lifeact (Figure 2 and Video 2). Apical targeting of aPKC began approximately ten minutes before NEB, when small, discontinuous aPKC foci began to appear, as previously reported (Oon and Prehoda, 2019). The interphase pulses of actin had no noticeable effect on these aPKC enrichments, suggesting that at this stage of the cell cycle, cortical actin dynamics are not coupled to aPKC movement (Figure 1C).

**Figure 2—video 1.** Videos used to calculate when apically directed aPKC and actin movements begin. aPKC-GFP expressed from its endogenous promoter (left panel) and mRuby-LifeAct expressed via insc-GAL4/UAS (middle panel) and merge (right panel with aPKC green and LifeAct magenta) are shown via a maximum intensity projection (MIP) constructed from optical sections through the front hemisphere of the cell. Each replicate is labeled with the larva and neuroblast number. Time (mm:ss) relative to NEB is shown in the upper left corner.

**Video 2.** Correlated dynamics of the Par protein aPKC and actin in a larval brain neuroblast. GFP-aPKC expressed from its endogenous promoter and the mRuby-Lifeact sensor expressed from the UAS promoter and *insc-GAL4* (drives expression in neuroblasts and progeny) are shown from simultaneously acquired optical sections with a maximum intensity projection of the front hemisphere of the cell.

While cortical actin and aPKC did not appear to be coupled during interphase, the two protein’s movements were highly correlated beginning in early mitosis (Figure 2 and Video 2). When cortical actin began flowing apically, the sparsely distributed aPKC patches that had accumulated on the cortex also began moving toward the apical pole. The transition to apically directed movement was nearly simultaneous for both proteins, although actin’s apical movement began slightly before aPKC’s (actin: 1.9 ± 1.0 min prior to NEB; aPKC: 1.3 ± 1.1 min; n = 13 neuroblasts with movies in Figure 2—video 1). Furthermore, while aPKC and actin both moved toward the apical cortex, actin dynamics occurred over the entire cortex whereas aPKC movements were limited to the apical hemisphere consistent with its specific targeting to this area (Figure 2 and Video 2). The continuous apical movements resulted in the concentration of both aPKC and actin at the apical pole. Interestingly, however, once aPKC was collected near the pole into an apical cap, it appeared to be static while cortical actin continued flowing apically. This phase of dynamic, apically directed actin with an apparently static aPKC apical cap continued for several minutes (e.g. until approximately 7:10 in Video 2). At this point actin and aPKC movements reversed, moving simultaneously toward the basal pole and the emerging cleavage furrow (Figure 2 and Video 2). We conclude that cortical actin and aPKC dynamics become highly correlated after interphase actin pulses transition to sustained, apically directed movements.

**Figure 3.**
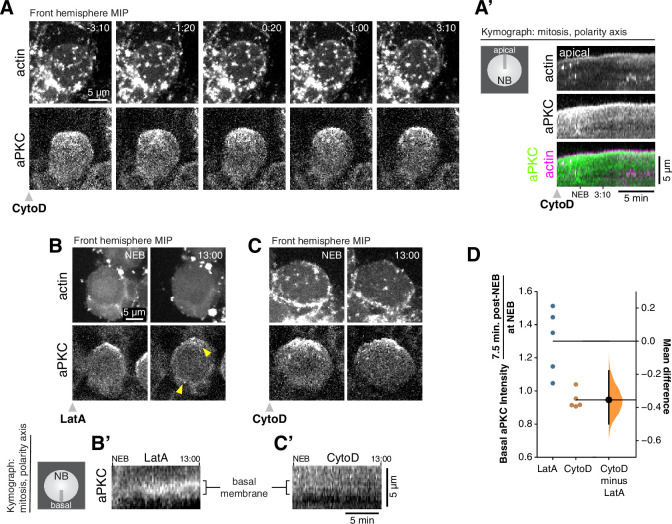
Cortical F-actin is required for aPKC coalescence and polarity maintenance. (**A**) Disruption of cortical F-actin dynamics using a low dosage (50 µM) of cytochalasin D (CytoD) causes immediate cessation of aPKC cortical movement. Selected frames from Figure 3—video 1 showing the inhibition of actin dynamics and accompanying loss of apically-directed aPKC movements. aPKC-GFP expressed from its endogenous promoter (‘aPKC’) and mRuby-LifeAct expressed via insc-GAL4/UAS (‘actin’) are shown via a maximum intensity projection (MIP) constructed from optical sections through the front hemisphere of the cell. (**A’**) Kymograph made from Figure 3—video 1 using a section of each frame along the apical-basal axis, as indicated. (**B**) Selected frames from Figure 3—video 3 showing significant entry of aPKC into the basal hemisphere (i.e. depolarization) following LatA treatment. Upper arrowhead highlights a localized enrichment of aPKC that is retained in the apical region. Lower arrowhead highlights increased aPKC in basal hemisphere. (**B’**) Kymograph made from Figure 3—video 3 using a section of each frame along the basal membrane near the cell’s equator as indicated. (**C**) Selected frames from Figure 3—video 4 showing the maintenance of aPKC polarization following CytoD treatment. (**C’**) Kymograph made from Figure 3—video 4 using a section of each frame along the basal membrane as indicated. (**D**) Gardner-Altman estimation plot of the fold increase in basal aPKC membrane signal following Latrunculin A (LatA) and CytoD treatments. The ratio of aPKC membrane signal on the basal membrane shortly after NEB to that at NEB is shown for individual LatA- and CytoD-treated neuroblasts (shown in Figure 3—video 5), along with the difference in means of the measurements. Statistics: bootstrap 95 % confidence interval (bar in ‘CytoD minus LatA’ column shown with bootstrap resampling distribution).

The correlation between actin and aPKC dynamics is consistent with our previous finding that depolymerization of actin with LatrunculinA (LatA) inhibits aPKC’s apically directed cortical movements (Oon and Prehoda, 2019). We further examined the relationship between F-actin dynamics and aPKC using low doses of Cytochalasin D (CytoD) that inhibit actin dynamics but maintain cortical structure (An et al., 2017; Mason et al., 2013). The apically directed movements of actin during early mitosis were inhibited by CytoD with the cortex rapidly becoming relatively static (Figure 3A and A’ and Figure 3—video 1). The loss of actin dynamics was immediately followed by cessation of apically directed aPKC movement such that it failed to form an apical cap (n = 11; neuroblasts shown in Figure 3—video 2). While both LatA and CytoD inhibited aPKC coalescence into an apical cap, we noticed that aPKC was maintained in the apical hemisphere for a longer period in CytoD-treated neuroblasts (Figure 3B–D, Figure 3—video 3, Figure 3—video 4, and neuroblasts used for measurements in Figure 3—video 5). Some localized enrichments remained in the apical hemisphere in neuroblasts treated with either drug, possibly due to their association with localized membrane enrichments, as recently reported (LaFoya and Prehoda, 2021). However, aPKC signal entered the basal hemisphere more rapidly in LatA- versus CytoD-treated neuroblasts (Figure 3D). Thus, aPKC appears to be better maintained in its polarized state when the cortical actin cytoskeleton remains intact (CytoD) compared to when it is completely depolymerized (LatA). We conclude that the cortical dynamics that drive the formation of the aPKC apical cap require the apically directed phase of actin dynamics that occurs during late prophase and metaphase. Furthermore, cortical actin structure may prolong the aPKC polarized state by slowing its diffusion into the basal hemisphere.

**Figure 3—video 1.** Inhibition of cortical actin and aPKC dynamics by Cytochalasin D. GFP-aPKC expressed from its endogenous promoter and the mRuby-LifeAct sensor expressed from the UAS promoter and *insc-GAL4* (drives expression in neuroblasts and progeny) are shown from simultaneously acquired optical sections with a maximum intensity projection of the front hemisphere of the cell. CytoD was added to the media surrounding the larval brain explant at 2 min prior to the beginning of the movie.

**Figure 3—video 2.** Movies used to quantify apical movements of aPKC in Cytochalasin D-treated neuroblasts. GFP-aPKC expressed from its endogenous promoter (left panel), the mRuby-LifeAct sensor expressed from the UAS promoter and *insc-GAL4* (middle panel), and a merge of the two channels (aPKC–green, LifeAct–magenta) are shown from simultaneously acquired optical sections with a maximum intensity projection of the front hemisphere of the cell. CytoD was added to the media surrounding the larval brain explant at 2 min prior to the beginning of the movie as indicated. Each replicate is labeled with the larva and neuroblast number. Time (mm:ss) relative to NEB is shown in the upper left corner.

**Figure 3—video 3.** Depolarization of aPKC induced by Latrunculin A. GFP-aPKC expressed from its endogenous promoter and the mRuby-Lifeact sensor expressed from the UAS promoter and *insc-GAL4* (drives expression in neuroblasts and progeny) are shown from simultaneously acquired optical sections with a maximum intensity projection of the front hemisphere of the cell. LatA was added to the media surrounding the larval brain explant at 2 min prior to the beginning of the movie.

**Figure 3—video 4.** Maintenance of polarized aPKC in a Cytochalasin D-treated neuroblast. GFP-aPKC expressed from its endogenous promoter and the mRuby-Lifeact sensor expressed from the UAS promoter and *insc-GAL4* (drives expression in neuroblasts and progeny) are shown from simultaneously acquired optical sections with a maximum intensity projection of the front hemisphere of the cell. CytoD was added to the media surrounding the larval brain explant at 2 min prior to the beginning of the movie.

**Figure 3—video 5.** Movies used to quantify maintenance of aPKC in Latrunculin A- and Cytochalasin D-treated neuroblasts. GFP-aPKC expressed from its endogenous promoter (left panel), the mRuby-LifeAct sensor expressed from the UAS promoter and *insc-GAL4* (middle panel), and a merge of the two channels (aPKC–green, LifeAct–magenta) are shown from simultaneously acquired optical sections with a maximum intensity projection of the front hemisphere of the cell. CytoD or LatA (as indicated) was added to the media surrounding the larval brain explant at 2 min prior to the beginning of the movie as indicated. Each replicate is labeled with the larva and neuroblast number. Time (mm:ss) relative to NEB is shown in the upper left corner.

### Correlated phases of cortical myosin II and actin dynamics

**Figure 4.**
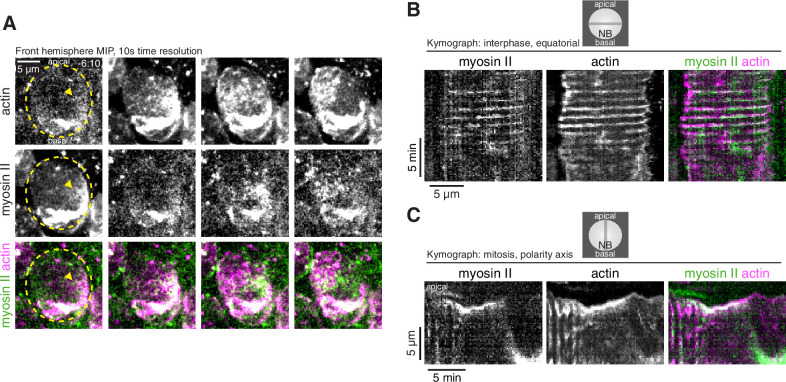
Dynamics of cortical actomyosin in asymmetrically dividing *Drosophila* larval brain neuroblasts. (**A**) Selected frames from Video 3 showing cortical actomyosin dynamics. GFP-Sqh expressed from its endogenous promoter (‘Myosin II’) and mRuby-LifeAct expressed via worniu-GAL4/UAS (‘actin’) are shown via a maximum intensity projection (MIP) constructed from optical sections through the front hemisphere of the cell. The outline of the neuroblast is shown by a dashed yellow line and arrowheads indicate the starting position of the cortical patches. Time is relative to nuclear envelope breakdown. (**B**) Kymograph constructed from frames of Video 3 during interphase using sections through the equatorial region of the cell as indicated. (**C**) Kymograph constructed from frames of Video 3 during mitosis using sections along the polarity axis of the cell as indicated.

The morphological changes in interphase cells (Figure 1D and Video 1) and cortical aPKC movements that were correlated with cortical actin dynamics in early mitosis (Figure 2 and Video 2), are consistent with a force generating process. While actin can generate force directly through polymerization, contractile forces are generated by the combined activity of F-actin and myosin II (i.e. actomyosin), and cortical pulsatile contractions of actomyosin have been observed in many other systems (Vicker, 2000; Munro et al., 2004; Michaux et al., 2018). The localization of myosin II in neuroblasts has been described as uniformly cortical or cytoplasmic in interphase and before metaphase in mitosis (Barros et al., 2003; Tsankova et al., 2017; Koe et al., 2018). We used rapid imaging of the full cell volume, simultaneously following a GFP fusion of the myosin II regulatory light chain Spaghetti squash (GFP-Sqh) with mRuby-Lifeact, to determine if myosin II dynamics share any of the cortical dynamic phases we observed for actin. For each phase of actin dynamics, we found that myosin II is localized to the cortex in a similar pattern (Figure 4 and Video 3), including during the apically directed continuous movements that polarize aPKC. Interestingly, however, the localization between the two was not absolute and there were often large cortical regions where the two did not colocalize in addition to the region where they overlapped (Figure 4 and Video 3). A similar pattern of overlapping cortical actin and myosin II localization has been reported in the polarizing worm zygote (Reymann et al., 2016; Michaux et al., 2018). Given the similarities in behavior of actin and myosin II, we conclude that the phases of cortical actin dynamics that occur during neuroblast asymmetric cell division include both actin and myosin II.

**Video 3.** Correlated dynamics of myosin II and actin in a larval brain neuroblast. GFP-Sqh (the myosin II regulatory light chain, Spaghetti squash) expressed from its endogenous promoter and the mRuby-Lifeact sensor expressed from the UAS promoter and *worniu-GAL4* (expressed in neuroblasts and their progeny) are shown from simultaneously acquired optical sections with a maximum intensity projection of the front hemisphere of the cell and the medial optical section. The neuroblast is highlighted by a dashed circle.

### Phases of cortical actomyosin dynamics coupled to neuroblast polarization, maintenance, and depolarization

**Figure 5.**
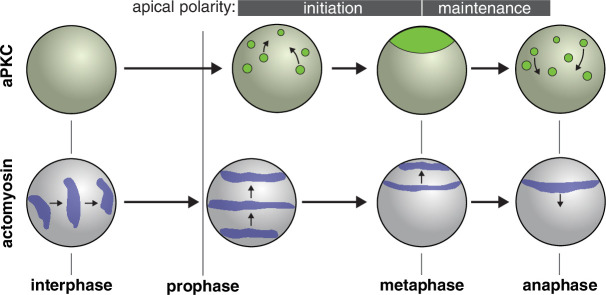
Model for role of actomyosin in neuroblast Par polarity. During interphase when aPKC is cytoplasmic, actomyosin pulsatile contractions are predominantly equatorial. During apical polarity initiation in prophase and shortly before when discrete aPKC cortical patches begin to coalesce, actomyosin transitions to more continuous movements directed toward the apical cortex. At anaphase apical actomyosin is cleared as it flows toward the cleavage furrow while the aPKC cap is disassembled.

Our results reveal previously unrecognized phases of cortical actomyosin dynamics during neuroblast asymmetric division, several of which coincide with the neuroblast’s cortical polarity cycle (Figure 5). During interphase, transient cortical patches of actomyosin undergo highly dynamic movements in which they rapidly traverse the cell cortex, predominantly along the cell’s equator, before dissipating and beginning a new cycle (Figure 1A). Shortly after mitotic entry the movements become more continuous and aligned with the polarity axis (orthogonal to the equatorial interphase pulses). The transition to apically directed cortical actin movements occurs shortly before the establishment of apical Par polarity, when discrete cortical patches of aPKC undergo coordinated movements toward the apical pole to form an apical cap. Importantly, cortical actin dynamics are required for aPKC to coalesce into an apical cap (Figure 3A and Figure 3—video 1). Apically directed actin dynamics continue beyond metaphase when apical aPKC cap assembly is completed (Figure 2), suggesting that actomyosin dynamics may also be involved in cap maintenance. A role for actomyosin in aPKC cap assembly and maintenance is supported by the lack of coalescence when the actin cytoskeleton is completely depolymerized (Oon and Prehoda, 2019), or when actin dynamics are inhibited but the cytoskeleton is left intact (Figure 3B and B’). The cycle of cortical actomyosin dynamics is completed when the movement abruptly changes direction at anaphase leading to the cleavage furrow-directed flows that have been previously characterized (Barros et al., 2003; Roubinet et al., 2017). While we have examined the relationship between actomyosin dynamics and cortical protein polarity, we note that a neuroblast membrane polarity cycle was recently discovered and found to require the actin cytoskeleton (LaFoya and Prehoda, 2021). The mechanical phases of the membrane polarity cycle may be related to the phases of cortical actomyosin dynamics we report here.

While cortical actomyosin dynamics had not been reported during neuroblast polarization, myosin II pulses have been observed in delaminating neuroblasts from the *Drosophila* embryonic neuroectoderm (An et al., 2017; Simões et al., 2017). The actomyosin dynamics reported here may be related to those that occur during delamination and provide a framework for understanding how actomyosin participates in neuroblast apical polarity. First, apically directed movements of actomyosin are consistent with the requirement for F-actin in the coalescence of discrete aPKC patches into an apical cap (Figure 3; Oon and Prehoda, 2019). How might cortical actomyosin dynamics induce aPKC coalescence and maintenance? In the worm zygote, pulsatile contractions generate bulk cortical flows (i.e. advection) that lead to non-specific transport of cortically localized components (Goehring et al., 2011; Illukkumbura et al., 2020). Whether the cortical motions of polarity proteins that occur during the neuroblast polarity cycle are also driven by advection will require further study.

The more rapid depolarization of aPKC in Lat- compared to CytoD-treated neuroblasts (Figure 3B–D), is also consistent with a potentially passive role for the actin cytoskeleton in polarity maintenance. Complete loss of the cortical actin cytoskeleton (LatA; Figure 3B) leads to more rapid entry of aPKC into the basal neuroblast membrane compared to when cortical actin dynamics is inhibited but the structure maintained (CytoD; Figure 3C). The difference could arise simply from an increase in cortical diffusion constant when the cortical actin mesh is removed. In this case, the actin cytoskeleton would participate in Par polarity via at least two mechanisms: by generating non-diffusive movements of polarity proteins through actomyosin-generated cortical flows (Figures 2 and 3), and by maintaining the polarized state by slowing the rate of diffusion (Figure 3).

## Materials and methods

**Key resources table:** 

Reagent type (species) or resource	Designation	Source or reference	Identifiers	Additional information
Genetic reagent (*Drosophila melanogaster*)	Lifeact-Ruby	Bloomington *Drosophila* Stock Center	BDSC:35545; FLYB:FBti0143328; RRID:BDSC_35545	FlyBase symbol: P{UAS-Lifeact-Ruby}VIE-19A
Genetic reagent (*D. melanogaster*)	insc-Gal4	Chris Doe Lab; Bloomington *Drosophila* Stock Center	BDSC:8751; FLYB:FBti0148948; RRID:BDSC_8751	FlyBase symbol: P{GawB}insc^Mz1407^
Genetic reagent (*D. melanogaster*)	aPKC-GFP	François Schweisguth Lab; Besson et al., 2015		BAC encoded aPKC-GFP
Genetic reagent (*D. melanogaster*)	wor-Gal4	Chris Doe Lab; Bloomington *Drosophila* Stock Center	BDSC:56553; FLYB:FBti0161165; RRID:BDSC_56553	FlyBase symbol: P{wor.GAL4.A}2
Genetic reagent (*D. melanogaster*)	Sqh-GFP	Royou et al., 2002		Expressed by natural *sqh* promoter
Chemical compound, drug	Latrunculin A	Sigma-Aldrich	Sigma-Aldrich: L5163	(50 µM)
Chemical compound, drug	Cytochalasin D	Enzo Life Sciences	Enzo Life Sciences: BML-T109-0001	(50 µM)

### Fly strains and genetics

UAS-Lifeact-Ruby (Bloomington stock 35545), BAC-encoded aPKC-GFP (Besson et al., 2015) and Sqh-GFP (Royou et al., 2002) transgenes were used to assess F-actin, aPKC and myosin II dynamics, respectively. Expression of Lifeact was specifically driven in nerve cells upon crossing UAS-Lifeact-Ruby to *insc-Gal4* (1407-Gal4, Bloomington stock 8751) or to *worniu-Gal4* (Bloomington stock 56553). The following genotypes were examined through dual channel live imaging: BAC-aPKC-GFP / Y; insc-Gal4, +/+, UAS-Lifeact-Ruby; and; worGal4, Sqh-GFP, +/+, UAS-Lifeact-Ruby.

### Live imaging

Third instar larvae were incubated in 30 ºC overnight (~12 hr) prior to imaging and were dissected to isolate the brain lobes and ventral nerve cord, which were placed in Schneider’s Insect media (SIM). Larval brain explants were placed in lysine-coated 35 mm cover slip dishes (WPI) containing modified minimal hemolymph-like solution (HL3.1). Explants were imaged on a Nikon Ti2 microscope equipped with a Nikon 60 × 1.2 NA Plan Apo VC water immersion objective, a Yokogawa CSU-W1 spinning disk, and two Photometrics Prime BSI Scientific CMOS cameras for simultaneous dual channel imaging. Explants expressing Lifeact-Ruby, and either aPKC-GFP or Sqh-GFP were illuminated with 488 nm and 561 nm laser light. Approximately 40 optical sections with step size of 0.5 µm were acquired throughout the neuroblast volume at time intervals of 10–15 s. For drug treatments, the culture media surrounding the explants were brought to final concentrations of 50 µM LatA (2 % DMSO) or 50 µM CytoD (0.5 % DMSO) at the start of the imaging session.

### Image processing, analysis, and visualization

Movies were analyzed using the ImageJ (via the FIJI distribution) and Imaris (Bitplane) software packages. To quantify transitions between cortical actin dynamic phases, we identified the frame when actin or aPKC began moving toward the apical pole persistently (for at least several minutes, unlike the interphase, pulsatile motions). Likewise, the start of basally directed actin flow was indicated by the initial frame when apical actin or aPKC moved persistently toward the basal hemisphere. To investigate the effects of LatA and CytoD on neuroblast cortical dynamics, we examined aPKC and actin signals in early prophase to first determine if actin dynamics had ceased (i.e. the drug had taken effect), which typically occurred within five minutes of treatment for both drugs. To measure the degree to which aPKC polarity was maintained, we quantified the basal intensity of aPKC (via a 3-µm-thick line scan) across the basal membrane of a single medial section at NEB and at 7.5 min after NEB. The fold change in basal intensity across time was calculated using the following equation, with the mean background intensity obtained from a featureless area outside the neuroblast:

$$FoldChange\in BasalIntensity=\frac{{\left(Meanbasalintensity-Meanbackgroundintensity\right)}_{NEB+7.5min}}{{\left(Meanbasalintensity-Meanbackgroundintensity\right)}_{NEB}}$$

## Data Availability

All data generated or analysed during this study are included in the manuscript and supporting files.

## Additional files

Transparent reporting form
